# The challenge of change in acute mental health services: measuring staff perceptions of barriers to change and their relationship to job status and satisfaction using a new measure (VOCALISE)

**DOI:** 10.1186/1748-5908-9-23

**Published:** 2014-02-20

**Authors:** Caroline Laker, Felicity Callard, Clare Flach, Paul Williams, Jane Sayer, Til Wykes

**Affiliations:** 1 Department of Psychology, Institute of Psychiatry, King’s College, PO77, Room 2.11, London Henry Wellcome Building, 16 De Crespigny Park, London, SE5 8AF, UK; 2 The University of Manchester, Oxford Road, Manchester, M13 9PL, UK; 3 South London & Maudsley NHS Foundation Trust, Bethlem Royal Hospital, Monks Orchard Road, Beckenham, BR3 3BX, UK

**Keywords:** Perception, Measures, Organisational change, Mental health, Nursing

## Abstract

**Background:**

Health services are subject to frequent changes, yet there has been insufficient research to address how staff working within these services perceive the climate for implementation. Staff perceptions, particularly of barriers to change, may affect successful implementation and the resultant quality of care. This study measures staff perceptions of barriers to change in acute mental healthcare. We identify whether occupational status and job satisfaction are related to these perceptions, as this might indicate a target for intervention that could aid successful implementation. As there were no available instruments capturing staff perceptions of barriers to change, we created a new measure (VOCALISE) to assess this construct.

**Methods:**

All nursing staff from acute in-patient settings in one large London mental health trust were eligible. Using a participatory method, a nurse researcher interviewed 32 staff to explore perceptions of barriers to change. This generated a measure through thematic analyses and staff feedback (N = 6). Psychometric testing was undertaken according to standard guidelines for measure development (N = 40, 42, 275). Random effects models were used to explore the associations between VOCALISE, occupational status, and job satisfaction (N = 125).

**Results:**

VOCALISE was easy to understand and complete, and showed acceptable reliability and validity. The factor analysis revealed three underlying constructs: ‘confidence,’ ‘de-motivation’ and ‘powerlessness.’ Staff with negative perceptions of barriers to change held more junior positions, and had poorer job satisfaction. Qualitatively, nursing assistants expressed a greater sense of organisational unfairness in response to change.

**Conclusions:**

VOCALISE can be used to explore staff perceptions of implementation climate and to assess how staff attitudes shape the successful outcomes of planned changes. Negative perceptions were linked with poor job satisfaction and to those occupying more junior roles, indicating a negative climate for implementation in those groups. Staff from these groups may therefore need special attention prior to implementing changes in mental health settings.

## Background

Health services are complex organisations that are characterised by substantial and ongoing organisational change as a result of research innovations and service developments. While there is common acknowledgement of the frequency of these changes, there has been little research that addresses how staff working in these services anticipate and respond to innovation [1,2], and none that focuses specifically on acute in-patient wards in the mental health services. An exploration of these issues in the mental health services is important because distinct social processes and contextual features exist here [3]. In particular, in-patients are affected either personally or vicariously by the Mental Health Act (2003), the legislation that allows enforced detention in the U.K, which can lead to enforced medication and conflict. These factors may present barriers to change that are setting-specific. In addition, mental health nurses on acute wards are also engaged in the social milieu of the ward for the duration of their shift with little time allocated for strategic planning, and are often expected to play a key role in delivering changes, making their role different from other health professions.

Previous research shows that organisational climate, or how staff perceive and respond to the characteristics of their work environment, affects work attitudes in mental health services [3-6]. A demoralising climate, as well as poorly orchestrated changes, may increase staff negativity and resistance to change as well as lowering morale [4]. It is also likely that nursing staff perceptions of implementation climate [7] may also affect other work attitudes. As observed by Kajermo et al., [8], many descriptive studies identify barriers to implementing evidence-based practice, but the strategic link between barriers, implementation and outcomes has yet to be adequately explored [3]. Despite nurses representing the majority of the workforce, perceptions of implementation climate have been under studied. The emotional responses of staff as a result of service changes have also had little attention. Understanding how staff perceive change may be of particular use since perceptions can influence the successful outcomes of changes [9].

We explore the relationships between staff perceptions of barriers to change and occupational status and job satisfaction to highlight how staff can be supported before and during processes of change, to improve the chances of successful implementation.

### Why has change been a challenge?

Mental health nurses perceive many objective barriers to change in acute ward settings, including limited resources (*e.g.*, beds, staff), the process of bed management, poor/indeterminate leadership, and violence [10,11]. Despite these perceptions, wide-scale changes to services have been pushed through in recent years, driven by political and economic factors. However, there is an increasing body of evidence in healthcare showing poor uptake of innovations and evidence-based findings into practice [12-14].

During periods of change, there are reciprocal interactions between the process and those involved [15]. The literature shows that low levels of involvement in change and changes that are not perceived to be beneficial can negatively impact the workforce. Organisational change in the UK National Health Service (NHS) is often imposed via a top down approach, which may not take into consideration the views of the majority of nursing staff. Indeed, change process issues such as poor involvement in planning, implementation and control of the project have been highlighted as potential barriers to success [16]. Staff in leadership roles may view changes more positively than those in direct care roles because they form part of the consultation process [17,18]. In our study, we will expand on this theme in a new context - acute mental health wards - hypothesising that nursing staff occupying managerial roles will view change more optimistically than more junior direct care staff.

In a study examining organisational justice, Haar et al., [18] found that those employees of a local government organisation who were likely to directly benefit from work-family policies viewed them more positively than those who were not affected, who saw them as unfair. We will develop this theme to illuminate how organisational justice is perceived in mental health settings.

Other studies linked to acceptance of change have been carried out [19]. In particular, ‘personal resilience,’ characterised by perceived control over proposed changes, optimism, and self-esteem, was a predictor of acceptance to change. Poor job satisfaction was an outcome for staff who were less open to changes (*i.e*., who perceive more barriers) [19], a finding that has been replicated in the wider healthcare literature [1,2]. Poor job satisfaction is not only damaging for the individual, but has organisational consequences as it has been linked to low staff retention [20]. We will explore the link between perceptions of barriers to change and job satisfaction in nursing staff in acute mental health settings.

Involving all staff in changes would present significant logistical problems. However, staff opinion might be accessed directly before changes are implemented using a measure that captures staff sentiment on the front line. In this study, we generate such a measure using a participatory method of measure development [21], which is known to improve participation and engagement. We believe that this participatory approach produces items that are of importance to the group under study [22,23].

### What are the issues in measuring change?

The findings from studies looking at private sector organisations or U.S. healthcare organisations may not be generalisable to U.K. healthcare organisations, which are funded through taxation and which have a constitutional aim to consider the best interests of the client group [24]. There are currently no psychometrically robust measures designed to capture nursing staff perceptions of barriers to change in acute in-patient wards, nor has the idea of staff’s direct participation in measure development been explored in this context. There are several measures developed in healthcare that focus on the uptake of evidence-based practice in clinical areas by mental health providers and nurses [25-32]. However, there are no measures which focus on general changes in a mental health setting.

In this study, we first develop a psychometrically robust measure specific to the context of acute mental healthcare that includes a qualitative exploration of the emotional responses of staff in relation to themes of organisational unfairness. Then we use the new measure to explore how characteristics identified in the organisational literature are linked to perceptions of barriers to change. Specifically, we explore: whether staff in more senior positions have more positive perceptions of barriers to change than staff working in direct contact with service users; and whether staff with negative perceptions of barriers to change also have poor job satisfaction. This will clarify the impact of change on nursing staff.

## Methods

### Ethical considerations

Ethical approval for this study was awarded by a local NHS Research Ethics Committee (07/H0809/49). Staff were assured that confidentiality and anonymity would be maintained unless any concerns about patient safety arose during the process. All staff gave informed consent to participate and were paid five pounds for their time.

### Measure development

**Figure 1:**
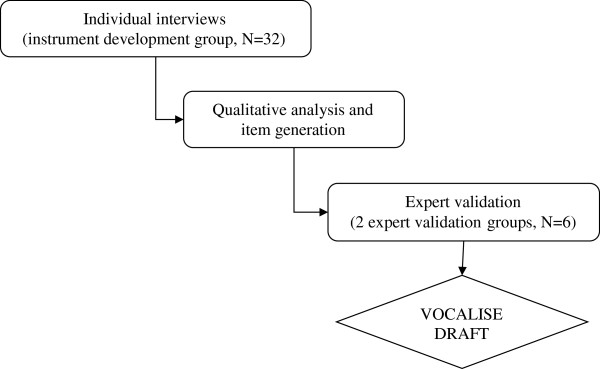
Qualitative phase: study design for instrument development and item generation.

The process of measure development occurred across three stages which are outlined in Figure 1.

### Qualitative phase: instrument development

The participatory framework for VOCALISE was adapted from the User-Focused Monitoring method developed by Rose [21] and Rose et al., [33,34]. This model is ideal because it promotes involvement from nurses as ‘stakeholders’ of their clinical areas. The core principle is to translate participant views into items to ensure strong item content validity. This is accomplished by maintaining a direct contextual link between the researcher and participant through shared experiences. This reduces the usual power differentials between interviewer and interviewee, resulting in data that more accurately represent participant views [35]. In this research, the shared experience was being a psychiatric nurse.

### Item generation

The criterion sample (N = 32) was drawn from a large London mental health trust and comprised eight individuals from each level of acute in-patient nursing staff (nursing assistants, entry level qualified nurses, senior nurses and ward managers).

Each staff member participated in a 30-minute interview based on themes drawn from the literature and from a reference group that included two nurses, a service user researcher, and a clinical psychologist. As the concept of ‘perceptions of barriers to change’ is abstract, the interviewee was asked to consider a scenario in practice where a significant change to clinical practice had occurred. Successive thematic analyses [36] were conducted using a qualitative software package (NVivo). This allowed new themes to be incorporated into subsequent interviews and ensured that the content domain was explored broadly, enhancing content validity.

Themes with the highest numbers of references and which were therefore the most important issues to staff were included as items that also made use of participants’ language. Consideration was also given to themes that were expressed with vehemence. Independent raters compared how similarly they interpreted the themes and codes of one interview using a function within NVivo to minimize subjectivity and increase how reliably the data were interpreted.

Two rounds of feedback from acute ward nursing staff were then used to improve the item wording. This expert validation process also formed another reliability check for the accuracy of the final questionnaire items and ensured high face validity. At the end of the development process, VOCALISE contained 23 items, which were answerable on a 6-point Likert scale ranging from strongly agree to strongly disagree.

### Psychometric testing

**Figure 2:**
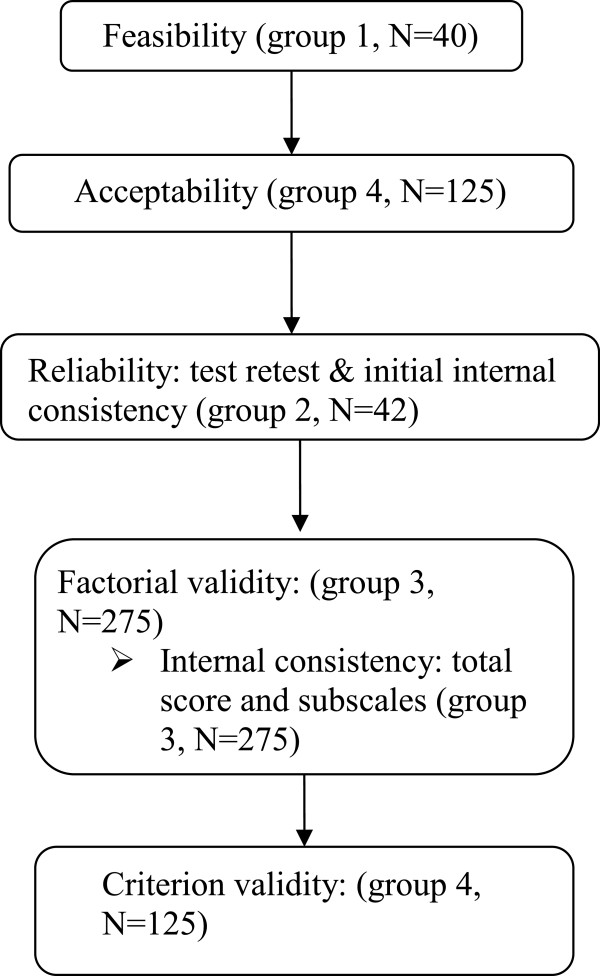
Quantitative phase: study design for psychometric testing.

Figure 2 outlines the psychometric testing process based on the Health Technology Assessment programme recommendations [37] and Streiner & Norman [38]. Analyses were conducted using STATA 11 and SPSS Statistics 20. The results of the psychometric tests were used to improve the measure, which meant that modifications occurred during the analyses and the final number of items was reduced.

### Sample and preliminary tests

All mental health acute ward nursing staff were invited to participate in this phase (ward managers, senior nurses, entry level qualified nurses and nursing assistants). Four sets of questionnaire data were collected from these staff, from all four boroughs of a large London trust. Each set of data was for a different purpose:

1. group one (N = 40): feasibility study

2. group two (N = 42): test-retest study

3. group three (N = 275): factor analysis and the internal consistency of any subscales

4. group four (N = 125): exploratory analyses of the associations between staff perceptions of barriers to change and occupational status and job satisfaction (criterion validity)

Preliminary tests included an intraclass correlation, which was computed to establish that ‘between ward’ variance was greater than ‘within group’ variance [5]. Random effects regression models, clustering on ward, were then used to explore the effects of all staff characteristics on VOCALISE. Random effects models were appropriate because the data were multi-level. Random effects models meet the assumption that differences in the data relate to subsets of the population (in this case, that groups were located within wards). They control for unobserved heterogeneity so that significant effects can be said to be true effects rather than those observed due to differences in the sample. This method of analysis was also appropriate because despite differences in the scores of individuals, within each ward, the distribution was roughly symmetrical, suggesting general consensus.

### Measures

Perceptions of job satisfaction were measured using the Index of Work Satisfaction (IWS) [39]. VOCALISE captured staff perceptions of barriers to change. Negatively phrased items were reverse scored so that higher scores indicated more negative perceptions.

### Assessing the psychometric properties of VOCALISE

Feasibility and acceptability were assessed on the 23-item measure. For feasibility, two additional questions assessing whether the measure was easy to complete and understand were asked of group one (N = 40), and for acceptability, a further two questions were added to the assessment of group four (N = 125).

### Reliability

Group two (N = 42) completed the questionnaire at two time points. Test-retest reliability was assessed on total scores using Lin’s concordance coefficient [40] and paired t-tests to investigate whether consistent bias existed. Item reliability was assessed using weighted kappa coefficients [41] on individual item scores at the two time points. Generally, scores of 0.21 to 0.4 are said to indicate ‘fair agreement’, scores of 0.41 to 0.60 indicate ‘moderate’ agreement, and scores above indicate ‘substantial’ agreement [42]. As data were often clustered on particular responses, this meant that a high kappa was not achievable irrespective of the level of concordance between the two time points. Therefore, kappa max was computed, which is the proportion of the maximum kappa attained [43]. The assessments were six to ten days apart, which was appropriate because the measure is of individual perceptions, which are subject to change over time. Items were removed on the basis of poor test-retest reliability.

The internal consistency of the scale using Cronbach’s alpha [44] was then investigated with items that had performed poorly on the basis of test-retest removed.

### Validity

Construct validity was examined through an exploratory factor analysis on responses from group three (N = 275), following preliminary checks (Bartlett’s test of sphericity and the Kaiser-Meyer-Olkin measure of sampling adequacy), to determine the degree of correlation in the data.

Using data from group three (N = 275), the internal consistency of the subscales were tested using Cronbach’s alpha [44].

Exploratory analyses of the effects of staff perceptions were undertaken to assess criterion validity on the data from group four (N = 125), using random effects regression models, clustering on ward, to predict VOCALISE total scores in relation to two hypotheses:

1. Staff perceptions of barriers to change are more positive in staff with higher organisational status [17]. The two groups were managerial staff (in the U.K., this includes ward managers and senior nursing staff) and direct care staff (in the U.K., this includes entry level qualified nurses and nursing assistants).

2. Staff with positive perceptions of barriers to change will also have high levels of job satisfaction [1]. The two groups were staff with positive perceptions of job satisfaction and staff with negative perceptions of job satisfaction.

The IWS scores were dichotomised to create the two groups, using an a priori point (171). This was the average from four studies of nurses’ job satisfaction, which used IWS [45-48]. Positive perceptions were therefore defined as scores of 171 or below.

## Results

### Qualitative phase: instrument development and item generation

There was a good spread across the demographic criteria of age, ethnicity, borough and gender of participants in this phase (see Table 1).

**Table 1:** Demographic characteristics of interview participants

		**Instrument development**
		**Group**
		**N = 32 (%)**
**Staff grade**	**Nursing assistants**	8 (25)
	**Entry level qualified nurses**	8 25)
	**Senior nurses**	8 (25)
	**Ward mangers**	8 (25)
**Ethnic group**	**White British/Other**	18 (56)
	*BME	14 (44)
**Gender**	**Male**	14 (44)
	**Female**	18 (56)
**Age**	**Mean (sd)**	38(9.29)
	**Range**	26 - 55

*Note. BME = black minority ethnic group.

During the interviews, staff focused on changes that were large scale and intended as long term. Examples include: the introduction of protected therapeutic interaction time for key workers and their clients, the introduction of the smoking ban in hospitals, and the introduction of the ‘Knowledge and Skills Framework’, which is an NHS framework designed to monitor and support the management of career development. Within these examples, all staff also described the effects of incremental local changes and daily ward changes.

Data reduction produced seven over-arching domains: ‘communication’, ‘generation of ideas’, ‘outcomes of changes’, ‘resistance’, ‘strategy’, ‘support and monitoring’, and ‘team dynamic’. Themes with fewer than 20 references were not included. Items were constructed around perceptions of the team/ward and perceptions of the self in relation to change. During the inter-rater reliability exercise, the raters achieved an 87% level of agreement in their interpretation of the main themes in the data. Those who participated in the expert validation process confirmed that the content of the measure was relevant and that the content domain had been widely explored.

The qualitative data illustrated the emotional responses of staff toward changes. Quotations that focus on the theme of organisational unfairness are presented in Table 2.

**Table 2:** Qualitative quotations on organisational unfairness

**THEMES**	**QUOTATIONS**
**STRATEGY: Not having a choice feels negative**	*Entry level qualified nurse:* ‘We felt it was an added burden we felt that we had no choice.’
	*Nursing assistant:* ‘Though I do think there was an element of sort of Machiavellian sort of style management going on but I don’t think he was being malicious and that’s why I probably why I didn’t complain if I felt that I was being really victimised then I would have done but I think he just had a very unrealistic idea about his project and I think he was you know his management style was quite you know quite sort of what the word is exactly patriarchal quite sort of authoritarian.’
**RESISTANCE: Patients don't want to**	*Nursing assistant:* ‘But like I say some patients just don’t want that interaction some patients are too poorly to have the interaction.’
	*Entry level qualified nurse:* ‘It came to a point where I think when most of the patients didn’t want to engage....Of course we couldn’t enforce them....So you go introduce yourself and say we’ll be meeting at such and such a point when the time comes and say well don’t I don’t want to talk about anything....Which makes it difficult....And very disheartening as well at times.’
**RESISTANCE: Unfair task allocation**	*Nursing assistant:* ‘people have said I went to university for three years if you expect me to make beds and clean..... You know I mean when you, you know you hear people saying oh about team, team work and all that you wouldn’t know team work if you were hit by Man United in a bus.’
	*Senior nurse:* ‘And the turnover of patients is quite high so there’s always – so you would catch up the way we do it now – you would catch up and see your people in the morning and say hello – how are you – is there anything on your mind that you badly need to talk about? So they’ll have like 5 or 10 minutes and that’s about it and everything else is directed to the unqualified to do which is sadly not the right thing to so because you should really spend a bit more time with your patient but that’s how – how we are doing it now.’
**RESISTANCE: Blame and resentment**	*Senior nurse:* ‘Yeah that’s it and then it’s difficult because the motivated people are like well why are we not doing this today and then you get that kind of lack of consistency....And I think then people get quite angry about that and yeah.’
	*Nursing assistant:* ‘And initially they, they were happy with this. And there wasn’t much resistance then it was when they actually seen us doing what we were doing. I think because we weren’t always working long days. Our initial shifts were four days a week it was I think it was half eight in the morning to half six. And, and I think people thought that that was not fair basically. So what it boils down people would be quite resentful and resist it in terms of, well – it’s jealous.’
	*Ward manager:* ‘I think that you know people automatically feel really dumped upon actually, and because, as well, there is another thing about people not really being aware of the bigger picture - I think so they just see it as well, I’m being asked to do my job plus run a group everyday…..’

### The psychometric properties of VOCALISE

#### Sample and preliminary tests

Demographic information from the four sets of questionnaire data collected from mental health acute in-patient nursing staff are described in Table 3.

**Table 3:** Demographic characteristics of participants

		**Group 1**	**Group 2**	**Group 3**	**Group 4**
		**N = 40 (%)**	**N = 42 (%)**	**N = 275 (%)**	**N = 125 (%)**
**Staff grade**	**Nursing assistants**	13 (32.5)	14 (33)	76 (27)	41 (33)
	**Entry level qualified nurses**	14 (35)	15 (36)	114 (41)	57 (46)
	**Senior nurses**	9 (22.5)	8 (19)	42 (16)	15 (12)
	**Ward mangers**	3 (7.5)	5 (12)	16 (6)	9 (7)
**Ethnic group**	**White British/Other**	16 (40)	15 (36)	88 (32)	33 (26)
	*BME	24 (60)	26 (62)	177 (63)	89 (72)
**Gender**	**Female**	19 (47.5)	20 (48)	159 (57)	78 (62)
	**Male**	21 (52.5)	22 (52)	110 (39)	46 (37)
**Age**	**Mean (sd)**	44.44 (9.07)	39.5 (8.8)	38.11 (9.83)	39.72 (10.01)
	**Range**	28 - 64	24 - 61	19 - 67	22 - 67

*Note. BME = black minority ethnic group.

A one-way analysis of variance assessing the effect of ward on the VOCALISE scores showed that the between group variance was greater than the within group variance, F(7, 114) = 3.23, p = 0.004; N = 122). This suggested that random effects models would be suitable.

In this sample, two demographic factors, age (Coef: -5.67, S.E: 2.57, p = 0.03, C.I: -10.70 to -0.64) and occupational seniority, significantly affected staff perceptions of barriers to change when assessed using the total score. These factors were therefore controlled in all following regression analyses where relevant.

#### Feasibility

The feasibility study (group one, N = 40) showed that VOCALISE was easy to complete (94% agreed) and easy to understand (100% agreed). All staff found that with minimal explanation the measure could be completed by self-report. Changes were made to item wording, and those with poor or loaded phrasing were rephrased using more neutral language. This was determined by the research team.

#### Acceptability

Of the 125 participants (group four): 84 (73%) thought the length of the questionnaire was about right; 23 (20%) enjoyed filling out the questionnaire, while 78 (67.8%) had neutral feelings; and 107 (93%) did not find completing the items upsetting.

The Flesch reading ease score for the measure was 62.4% (the recommended score for standard documents is between 60% and 70%). The Flesch-Kincaid Grade Level score was 7.6 (the recommended score is between 7.0 and 8.0). No items were altered as a result of the acceptability study.

#### Reliability

Test-retest reliability was assessed using data from group two (N = 42). Four items were unreliable with a kappa max below 0.39 and were dropped from the scale, leaving eight items with fair reliability (0.39 to 0.49), seven items with moderate reliability (0.50 to 0.56), and three items with substantial reliability (0.61 to 0.71).

Concordance between the total scores was good (Total score, rho = 0.76). However, a paired *t*-test showed that there was a significant difference between the two time points (t = -2.10; p = 0.04; mean difference = -2; 95% C.I: -3.93 to -0.07). Test-retest reliability was therefore assessed according to staff group revealing that staff in direct care roles (N = 26) were likely to change their scores (t = -2.91; p = 0.008; mean difference = -3.12; 95% C.I: -5.32 to -0.91). The scores of those in managerial roles were stable (t = 0.35; p = 0.73; mean difference = 0.64; 95% C.I: -3.36 to 4.64; N = 11). The test retest reliability of the ‘I’ statements and general statements was also assessed showing that over time, the general statements (t = -1.88; p = 0.07; mean difference = -1.24; 95% C.I: -2.59 to 0.10; N = 37) were more likely to change than the ‘I’ statements (t = -1.50; p = 0.27; mean difference = 0.14; 95% C.I: -1.82 to 0.27; N = 40).

Cronbach’s alpha [44] was used to test the internal consistency of the 19-item scale. The overall alpha was 0.71, indicating acceptable reliability [49]. However, one item was dropped from the scale because it was poorly correlated with the total, indicating poor internal consistency, leaving a final alpha of 0.75, and 18 remaining items. These items are presented in Table 4.

**Table 4:** VOCALISE measure

***NO.***	***ITEM***
1.	When it comes to change, information is not circulated effectively on my ward.
2.	I feel confident when delivering new changes.
3.	My whole team is regularly consulted about new ideas for ward practices.
4.	I’m too busy to keep up to date with information about the changes that are happening on my ward.
5.	We can easily fit new changes in with our usual ward practices.
6.	I feel disheartened when others do not want to get involved in changes.
7.	I think that managing risk is more important than delivering new changes.
8.	Changes just increase my workload and make my life harder.
9.	It is not clear how all changes that we are asked to make will really benefit my ward.
10.	My teammates think that there is no point trying to implement some changes because they won’t work.
11.	I find it de-motivating when new changes do not take patients’ wishes into account.
12.	I think that some staff would rather let others take the lead in making changes.
13.	When some staff stop engaging with planned changes resistance spreads through my whole team.
14.	I do not really understand how to deliver some of the changes that are suggested by the management.
15.	Changes are audited to increase their consistent delivery on my ward.
16.	I always challenge team members who are avoiding delivering new changes.
17.	Inadequate staffing prevents changes being successful on my ward.
18.	Poor leadership prevents changes happening on my ward.

#### Validity

The VOCALISE measure was then subjected to an exploratory factor analysis to determine whether the patterns of relationships amongst the items could be simply explained by more than one underlying construct [50]. This assessment was carried out on the 18-item measure, using data from group three (N = 275; fully completed N = 240). The correlation matrix showed weak/moderate correlations in all items. Bartlett’s test of sphericity was significant (p = <0.001), which suggested that the variables were adequately correlated for a factor analysis. The Kaiser-Meyer-Olkin measure of sampling adequacy was good (0.8).

As part of the exploratory process of factor analysis, solutions were analysed using both orthogonal rotation using Varimax and oblique rotation using Promax, and included five, four, three and two factors. An oblique solution was preferred because it showed the clearest differentiation between factor loadings. In this solution, three factors were retained because they fell before a steep drop in the curve of the scree test [51]. The first 3 factors accounted for 43.50% of the variance and the eigen values were 4.23, 1.99 and 1.61

The item groupings from the factor analysis had adequate internal consistency [49]. Subscale one, characterised by a sense of ‘powerlessness’, had an overall alpha of 0.73; N = 262. Subscale two, which is suggestive of ‘confidence,’ had an overall alpha of 0.66, N = 255, and subscale three, which echoes feelings of ‘de-motivation’, had an overall alpha of 0.59, N = 261.

In order to aid interpretation, ranges were developed to show whether the VOCALISE total and subscale scores were positive or negative. These ranges are described in Table 5. Ambivalence in this case indicates that staff found it difficult to decide between slightly agree and slightly disagree.

**Table 5:** Interpretative ranges for VOCALISE

**Scale**	**Items (total)**	**Positive range**	**Negative range**	**Ambivalent range**	**Midpoint**
Total scores	1-18 (18)	18 - 54	72 - 108	55 - 71	63
Powerlessness	4,5,7,8,9,14,17 (7)	7 - 21	28 - 42	22 - 27	24
Confidence	1,2,3,10,15,16 (6)	6 - 18	24 - 36	19 - 23	21
De-motivation	6,11,12,13,18 (5)	5 - 15	20 - 30	16 - 19	17.5

Exploratory analyses of the associations between VOCALISE and occupational seniority and job satisfaction were then conducted to assess criterion validity. In group four (N = 125), the mean VOCALISE score was: 62.24 (sd = 11.47; range 38 to 93).

#### Occupational status

Occupational status was significantly associated with staff perceptions of barriers to change after controlling for age (Coef: -6.62, S.E: 2.59, p = 0.01; C.I: -11.69 to -1.55, N = 108, 8 wards). The predicted mean VOCALISE score in the managerial group was 56.72, and 63.34 in the direct care providers group.

#### Job satisfaction

Those with negative perceptions of the barriers to change also had poor job satisfaction after controlling for age and occupational status (Coef: 10.43, S.E: 1.97, p = 0.001; C.I: 6.58 to 14.30, N = 101, 8 wards). The predicted mean VOCALISE score in the high job satisfaction group was 56.76, and the predicted mean score in low job satisfaction group was 67.20.

## Discussion

### Measuring perceptions of barriers to change

VOCALISE emerged out of a novel method of measure development that emphasizes the participation of nursing staff as a stakeholder group within mental health settings. As a brief self-report tool, VOCALISE can be used to capture staff perceptions of large scale changes to practice, concisely. VOCALISE is advantageous over previous measures (such as OSC, ORC, BARRIERS, EPBAS) because it combines aspects of the organisational, social/emotional and psychological context with staff perceptions of barriers to change, rather than treating each construct in isolation [3,25,26,52,53]. This type of measure might therefore usefully inform decisions for innovation made at the organisational level, by highlighting the negative impact of change on individuals.

The participatory method yielded items that were descriptive and evaluative, suggesting that both are important in allowing staff to express cognitive and affective responses to change. Further, the benefits of the participatory model used to develop VOCALISE are visible in both the breadth of the construct investigated and the rich content of the items. The scale addresses barriers arising from ward/environmental factors as well as from social factors. The content of some items (*e.g*., eight, nine and eleven – see Table 4) are of interest because they exemplify feelings of organisational unfairness relating to work pressures, which appear to have arisen as a result of poor consultation around new changes. Although the idea of work pressure as a barrier to organisational readiness for change is not new [53], VOCALISE makes the link between work pressure and the way that innovation is carried out explicit, thereby specifically addressing perceptions of implementation climate. These more focused features may have been missed using a non-participatory method, and item 11, which addresses nurses’ perceptions about the effects of change on service users, is a new development. The links between how staff experience change and the feasibility of new changes in clinical practice may therefore be exposed successfully using this type of method.

The psychometric properties of the final measure were promising. VOCALISE showed acceptable agreement, according to Cohen’s kappa [41], and the total score showed good test-retest concordance. A total of 13 staff (35%) altered their score by more than 5 points in the direct care group but not in the managerial group, which indicates their sensitivity to instability in the inpatient environment in mental healthcare rather than rater bias. Indeed, during the qualitative phase of this study, staff suggested that their views might alter depending on the day, the staff on shift, and the client group. In practice, this suggests that VOCALISE data should be collected during a prescribed time frame, and the score should represent an aggregate of perceptions during that time.

Researchers in the field of implementation science have argued that items capturing ‘implementation climate’ should not be value-based and should comprise the shared perceptions of the workforce using descriptive, rather than evaluative items that focus on the organisations’ implementation practices and policies [7,54]. Using descriptive statements that focus on the team rather than the individual might increase within group consensus [7], because individuals’ views of themselves are likely to vary more than individuals’ views of the team. However, in an environment where both positive and negative approaches to implementation exist, the use of purely descriptive statements is a challenge [55]. The participatory method used to develop VOCALISE generated eight ‘I’ and ten general statements containing the issues that staff referenced most frequently and therefore felt to be the most important when it came to describing the barriers to delivering significant changes to practice.

How statements of perception function as a means of measurement in an environment that is subject to dynamic changes on a daily basis is worthy of further clarification. A mixture of ‘I’ statements as well as general statements may be of use because, as this study shows, ‘I’ statements are less likely to change over time. There were also less missing data in the ‘I’ statements, perhaps because staff found these items easier to answer. Further, despite a mixture of both descriptive and evaluative statements, aggregated scores were successful in reflecting a ward view, because there was higher consensus in each group than between groups. The internal consistency of the entire scale was good, and the internal consistency of the subscales was adequate.

### What are the components of perceptions of barriers to change?

The exploratory factor analysis indicated three latent psychological dimensions. Factor one links unsuccessful change to components of acute ward working that staff perceive as beyond their control. This highlights a sense of powerlessness that may be linked to poor work-related autonomy or to how staff prioritise change. Factor two appears to relate to staff confidence in how the process of change is managed. Factor three is characterised by a sense of disillusionment and loss of motivation that impacts on the team dynamic, perhaps creating a barrier in group commitment to the process of change.

### Are poorer perceptions of barriers to change related to other work issues?

Other studies have shown that staff with higher organisational status [18,56] view changes more favourably than those in more junior positions. Further, job satisfaction has been linked to staff who express more open views about new changes [1]. These results were replicated with VOCALISE, which demonstrates criterion validity.

Issues of consultation and fairness in relation to changes may be linked to organisational justice theory, which suggests that managerial staff perceive change more favourably because of the way that organisational outcomes are determined [56]. Staff in leadership roles are more likely to be involved in the planning stages of new changes and therefore have an increased sense of control and responsibility over them. The qualitative findings showed that direct care staff frequently described poor involvement in new changes, which supports previous findings in the literature [17,18]. A lack of interest in innovation existed amongst the client group, which affected staff motivation to deliver some changes. This increased feelings of blame and resentment among the team, and also emerged in response to issues of unfairness as linked to innovation. In the qualitative data, this theme was most frequently referenced by nursing assistants.

As the qualitative data identified, the direct care staff also made links between powerlessness and feasibility issues, feeling a sense of injustice because they were not able to deliver changes if neither their clients nor they perceived much benefit. Emotional responses such as blame and resentment also emerged in response to unfair task allocation, or low commitment to change by colleagues. This may have ramifications for the quality of care delivered as those in direct care roles are generally those who deliver changes and who spend the most time with patients.

More research is therefore required to explore whether emphasising the involvement of nursing staff in such changes might prove an effective method of managing changes more successfully.

Other studies of staff views of organisational changes have emphasised psychological concepts such as work related empowerment [1]; control over proposed changes [19,57]; motivation [1]; and levels of optimism and self-esteem, which have been combined into ‘resilience’ [19,57]. Context-specific variables that capture information about how staff regard the change process, such as the provision of information, staff involvement and social support, have also been found to influence openness to change [16,56]. As a measurement tool, VOCALISE is novel because it incorporates many of these concepts into one measure (powerlessness over proposed changes, confidence/optimism and de-motivation). The individual items identify barriers/openness to change and perceptions relating to the change process as well as context specific issues.

### Limitations and future work

VOCALISE was designed for use in a randomized controlled trial to implement nurse-led psychological therapies on acute in-patient wards, to assess how staff respond to general changes. Although this was a project to implement evidence-based practice, VOCALISE was not designed to rate clinician responses to the importance and relevance of evidence-based practice, specifically.

The data for this study were collected within one mental health trust. This was a very large service covering different demographic and geographically diverse areas, but this still limits generalisability.

As the amount of variance explained by the factors in this study was low and some factor loadings were relatively low, a confirmatory factor analysis on a larger sample would be useful to confirm the measure structure.

### Implications

The participatory method used to construct VOCALISE revealed items which staff felt to be important. When considering complex concepts such as staff responses to innovation, this method proved effective in illuminating both staff perceptions of barriers to change but also some of the psychological and emotional effects of change on staff. This stakeholder-focused method of measure development has potential in other areas of implementation science where the relationship between change and those involved are integral to the successful uptake of change. It may, for example, usefully illuminate service user responses to innovation or to assess the barriers in sustaining innovation.

Although VOCALISE was developed to capture staff responses to significant, general changes in mental health settings, it could also measure local and project-based change. The scale was not specifically designed to assess incremental change, but the emotional and psychological aspects of the scale also reflect how staff experience the ongoing nature of change in the NHS, and therefore some effects of incremental change might be usefully picked up by VOCALISE. The item content of VOCALISE could be generalisable to other health settings, but more work should be undertaken to assess how VOCALISE performs in different areas.

The findings suggest that those members of the nursing workforce who are in direct care positions are ambivalent about new changes. This may explain why changes are difficult to embed, and staff motivation, confidence and feelings of ownership over new changes should be addressed to improve uptake.

## Conclusions

VOCALISE might be used in future research to identify whether nursing staff are motivated and confident enough to deliver new changes, as well as providing an opportunity for staff to engage with the process of change. As each of the factors may predict whether change is likely to be successful, interventions may then be tailored to improve the likelihood of embedding of changes into existing systems and practices. If required, strategies may be introduced to help embed new changes. A measure of this type could also allow the impact of changes on staff over time to be studied, which is important as low morale and negative perceptions of the workplace will be detrimental to the quality of care provided.

## Competing interests

This article presents independent research funded by the National Institute for Health Research (NIHR) under its Programme Grants for Applied Research scheme (RP-PG-0606-1050). The views expressed in this publication are those of the authors and not necessarily those of the NHS, the NIHR or the Department of Health.

## Authors’ contributions

CL carried out the data collection, analyses and drafted the manuscript. TW, FC, and JS provided expert advice during the qualitative phase. CF and PW provided statistical advice. TW conceived of the study, participated in its design and coordination, and was the chief investigator. TW, FC and JS helped to draft the manuscript. All authors read and approved the final manuscript.
